# Modulation of AMPA receptor mediated current by nicotinic acetylcholine receptor in layer I neurons of rat prefrontal cortex

**DOI:** 10.1038/srep14099

**Published:** 2015-09-15

**Authors:** Bo Tang, Dong Luo, Jie Yang, Xiao-Yan Xu, Bing-Lin Zhu, Xue-Feng Wang, Zhen Yan, Guo-Jun Chen

**Affiliations:** 1 Department of Neurology, the First Affiliated Hospital of Chongqing Medical University, Chongqing Key Laboratory of Neurology, 1 Youyi Road, Chongqing 400016, China; 2 The People’s Hospital of Anyue County, 68 Wai-Nan Street, Anyue County, Si-Chuan Province, 642350,China; 3 Department of Physiology and Biophysics, State University of New York at Buffalo, Buffalo, NY, 14214, USA

## Abstract

Layer I neurons in the prefrontal cortex (PFC) exhibit extensive synaptic connections with deep layer neurons, implying their important role in the neural circuit. Study demonstrates that activation of nicotinic acetylcholine receptors (nAChRs) increases excitatory neurotransmission in this layer. Here we found that nicotine selectively increased the amplitude of AMPA receptor (AMPAR)-mediated current and AMPA/NMDA ratio, while without effect on NMDA receptor-mediated current. The augmentation of AMPAR current by nicotine was inhibited by a selective α7-nAChR antagonist methyllycaconitine (MLA) and intracellular calcium chelator BAPTA. In addition, nicotinic effect on mEPSC or paired-pulse ratio was also prevented by MLA. Moreover, an enhanced inward rectification of AMPAR current by nicotine suggested a functional role of calcium permeable and GluA1 containing AMPAR. Consistently, nicotine enhancement of AMPAR current was inhibited by a selective calcium-permeable AMPAR inhibitor IEM-1460. Finally, the intracellular inclusion of synthetic peptide designed to block GluA1 subunit of AMPAR at CAMKII, PKC or PKA phosphorylation site, as well as corresponding kinase inhibitor, blocked nicotinic augmentation of AMPA/NMDA ratio. These results have revealed that nicotine increases AMPAR current by modulating the phosphorylation state of GluA1 which is dependent on α7-nAChR and intracellular calcium.

Nicotinic acetylcholine receptors (nAChRs) in the central nervous system participate in diverse functions, such as learning and memory, reward and drug abuse^1^,^2^. Emerging evidence demonstrates that nAChRs might be acting in the prefrontal cortex (PFC) to influence these cognitive functions^3^,^4^. In addition to their important role in neurotransmitter release and synaptic plasticity^5^,^6^, nAChRs layer specifically regulate neuronal excitability in deep layers of PFC, which has been recently demonstrated by Poorthuis and colleagues^7^. They showed that nicotine causes inhibition of layer II/III pyramidal neurons and activation of both interneurons and pyramidal neurons in layer V/VI, resulting in a net enhancement of output neuronal activity^7^.

Layer I receives input from almost all types of afferent fibers that reach the neocortex. Neurons in this layer exhibit the highest concentration of dendritic terminals of neocortex and form extensive synaptic neuropils interconnecting neurons in deep layers^8^,^9^,^10^, suggesting that layer I might be important in the PFC context. It is known that almost all neurons in this layer are GABAergic and can be depolarized by nAChRs^8^,^11^. In addition, acetylcholine activation of these neurons is inhibited by nAChR antagonists^11^,^12^,^13^, indicating that nAChRs play a key role in cholinergic regulation of neuronal activity in layer I.

nAChRs can regulate synaptic plasticity in layer I by increasing the spontaneous EPSC^14^. This may be achieved by enhancing AMPA receptor (AMPAR) mediated current which has been demonstrated in dopaminergic neurons^15^. In contrast to AMPAR, the NMDA receptor (NMDAR) is inactivated by nicotine in layer V pyramidal neurons in PFC^16^, suggesting that these glutamatergic receptors might be differentially regulated by nAChR in a region specific manner. However, how nAChRs might regulate the function of AMPAR and/or NMDAR in layer I remains unclear.

In this study, we assessed nicotine effect on AMPAR- and NMDAR- mediated currents in layer I neurons, and identified the potential subunits of nAChR and AMPAR that might be involved in this regulation. Results showed that activation of nAChRs led to the selective enhancement of AMPAR mediated current, which was dependent on α7-nAChR, intracellular calcium and the phosphorylation state of AMPAR subunit GluA1.

## Materials and Methods

### Slice preparation

All protocols were approved by the Commission of Chongqing Medical University for ethics of experiments on animals and were conducted in accordance with international standards. Male Sprague-Dawley (SD) rats (2weeks) were obtained from the Experimental Animal Center of Chongqing Medical University. Brain slices were prepared as previously reported^17^,^18^,^19^. Briefly, SD rats were anesthetized with 10% chloralic hydras (300 mg/kg). PFC slices (350 μm) were prepared with a Leica (Germany) VP1200S Vibratome and then incubated in artificial CSF (ACSF, in mM: 119 NaCl, 26NaHCO_3_, 2.5 KCl, 1MgCl_2_,1.25NaH_2_PO_4_,2CaCl_2_ and 25 glucose, pH 7.4, 310 mOsm) at room temperature (25 °C) bubbled with 5% CO_2_ and 95% O_2_ for at least 1 hr before recording.

### Patch clamp recordings

Whole-cell recording was performed as described previously^20^,^21^. Glass microelectrodes (Sutter, USA) were shaped by a pipette puller (P-97, Sutter, USA) with a resistance of 3–5 MΩ when filled with internal solution. The liquid junction potential was around −10 mV, which was corrected before sealing. A Multi-clamp 700B amplifier (Axon, USA) was used for the recordings. Signals were sampled at 10 kHz and filtered at 2 kHz. A stable baseline was obtained for at least 5 min before experiments and data were discarded when the access resistance (15–20 MΩ) was changed by 20% at the end of recording.

The evoked EPSC currents were generated with a 40μs pulse (0.1 Hz) from a stimulation isolation unit controlled by an AMPI generator (Master-8, USA). A bipolar stimulation electrode (FHC) was positioned ~50μm rostral to the recording electrode in the same layer^16^. The internal solution contained (in mM):130Cs-methanesulfonate, 10HEPES, 10CsCl, 4NaCl, 1MgCl_2_, 1EGTA, 5NMG, 5MgATP, and 0.5Na_2_GTP and 12 phosphocreatine, pH 7.2, 275–290 mOsm. Bicuculine(10 μM) was added to the bath solution to block GABA_A_ receptors. The evoked currents at −70 mV were identified as the AMPAR-mediated currents. Neurons were then voltage clamped at +40 mV, and the amplitude of the evoked EPSC 50 ms post-stimulus was considered as the NMDAR-mediated currents^22^,^23^. AMPA/NMDA ratio was calculated by AMPAR-mediated current (AMPA current) relative to NMDAR-mediated current (NMDA current). For the pure NMDA current recording, AMPAR antagonist CNQX (20 μM) and bicuculine(10 μM) were added in the ACSF when cells were voltage-clamped at +40 mV. For paired-pulse ratio recording, a paired-pulse protocol of two stimuli at an inter pulse interval of 50 ms was applied while the cells were voltage-clamped at −70 mV. Paired-pulse ratio (PPR) was defined as the second peak amplitude (P2) divided by the first peak amplitude (P1).

For the miniature EPSC (mEPSC) recording, 1 μM TTX, 10 μM bicuculine and 50 μM APV was applied to ACSF to block voltage-gated sodium channels, GABA_A_ and NMDA receptors, respectively. The pipette solution contained (in mM) 140 potassium gluconate, 5 KCl, 10 HEPES, 0.2 EGTA, 2 MgCl_2_, 4 MgATP, 0.3 Na_2_GTP, and 10 Na_2_-phosphocreatine, pH7.2 with KOH.

For the measurement of inward rectification of AMPA current, spermine (100 μM) was added in the internal solution, while bicuculine (10 μM) and APV (50 μM) were added in ACSF. AMPARs are heterotetramer composed of GluA1-A4 (also termed GluR1-R4). The GluA2 lacking (containing GluA1) receptors are permeable to Ca^2+^ that can be blocked by polyamines such as spermine at positive potentials, thus resulting in a characteristic inwardly rectifying current-voltage (I-V) relationship^24^,^25^,^26^. Neurons were held at −60, −40, −20, 0, +20 or +40 mV, respectively. The resultant inward rectification (IR) was calculated by dividing the absolute amplitude of average EPSC at −60 mV by that at +40 mV^15^,^26^.

### Reagents

The following reagents (final concentration) were included in this study. DHβE (1 μM), MLA (10 nM), APV(50 μM), IEM-1460(50 μM), Ro318220(10 μM), Bicuculine (10 μM), TTX(1 μM) were from TOCRIS. (−)-nicotine (N3876, covered with dark paper when in use), U0126 (10 μM), FK506 (10 μM), KN62 (15 μM), H89 (10 μM), BAPTA (10 mM) and spermine (100 μM) were from Sigma. Lipid solvent were made in stock solutions in DMSO (1:1000–2000), while the same dilution of DMSO were used in control solutions. Peptides that were designed to block the phosphorylation of GluA1 at the PKA (S845), CAMKII (S831) and PKC (S816/818) sites were synthesized and purified by Pepmic Co., Ltd (Suzhou, China). The sequences of PKA S845 was TLPRNSGAG (-SGAG), scrambled control was NRPGGTLSA (-TLSA). That of CaMKII S831 was QSINEAIRTSTLPRN (-LPRN), scrambled control was NLIITEQRPSSNART (-NART). And that of PKCS816/818 was EFCYKSRSESKRMKGFC (-KGFC), scrambled control was RGMKESSKKCCRSFFYE (-FFYE). They were all diluted in internal solution with a final concentration of 200 μM.

### Data analysis

Ten consecutive traces of evoked EPSC current were averaged to reach a final value. All values were expressed as mean ± SEM. Paired Student’s t-test was used for comparing nicotine effect in the same cells before and after application. Two way ANOVA and post-hoc testing was used to compare different groups. Mini Analysis program (Synaptosoft, Leonia, NJ) was used to analyze mEPSC amplitude and frequency. Individual synaptic events with fast onset and exponential decay kinetics were captured with threshold detectors in Mini Analysis software.

## Results

### Nicotine selectively increased AMPA current

**Figure 1:**
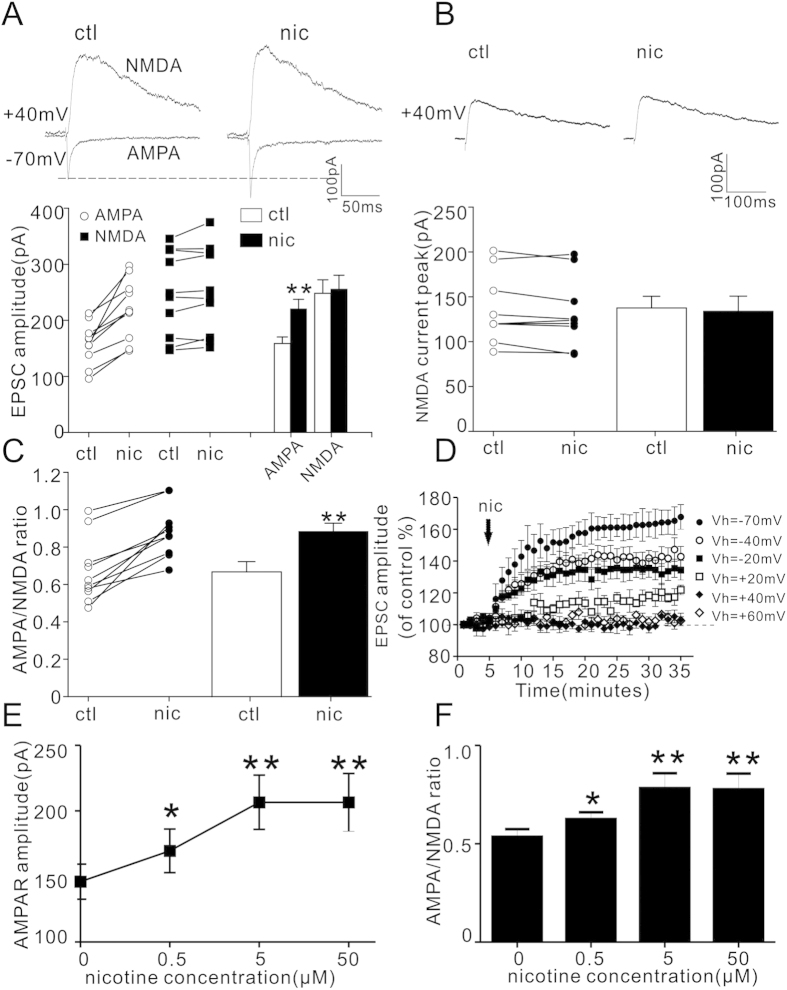
Nicotine increases AMPA/NMDA ratio but not NMDA current in PFC layer I neurons. (**A**) Representative traces of evoked EPSC at +40 mV (mixed NMDA) and −7 0mV (AMPA) in the absence (ctl) and presence (nic) of nicotine (5 μM) are shown on the top; Dot plots and histograms summarizing the amplitude of AMPA current and NMDA current are shown on the bottom. The amplitude of NMDA current was measured at 50ms post-stimulus at +40 mV. **P < 0.001 (nicotine vs. control in the same cell, paired Student’s t-test, n = 10). (**B**) Representative traces of NMDA current (in the presence of 20μM CNQX and 10μM bicuculine) at +40 mV before (ctl) and after nicotine (nic) are shown on the top; Dot plots and histograms summarizing the peak amplitude of NMDA current from top traces are shown on the bottom (P = 0.566, nicotine vs. control in the same cells, paired Student’s t-test, n = 8). (**C**) Dot plots and histograms of APMA/NMDA ratio from A before (ctl) and 10 min after nicotine (nic, 5 μM) application (**P < 0.001, nicotine vs. control in the same cell, paired Student’s t-test, n = 10). (**D**) Time course of the evoked EPSC amplitude before and after nicotine (5 μM) at holding potentials of −70 (n = 7), −40 (n = 5), −20 (n = 5), +20 (n = 5), +40 (n = 5) and +60 mV (n = 5), respectively. Nicotine causes vary degrees of current enhancement at these holding potentials, except at +40 and +60 mV. (**E,F**) Dose-response relationship of AMPA current (**E**) and AMPA/NMDA ratio (**F**) at nicotine concentration of 0, 0.5, 5 and 50 μM, respectively. *P < 0.05, **P < 0.01 (nicotine vs. control in the same cells, paired Student’s t-test, n = 7).

We have previously reported that nicotine increases the amplitude and frequency of sEPSC in layer I neurons of PFC^14^. To identify the potential role of AMPAR and NMDAR, we first recorded AMPA and NMDA currents in the presence of nicotine. The amplitude of AMPA current, NMDA current and AMPA/NMDA ratio before nicotine treatment were 158.27 ± 11.79 pA, 247.92 ± 24.08 pA and 0.67 ± 0.05, respectively. Then, nicotine (5 μM) was bath applied in ACSF for 10 minutes^14^,^27^,^28^. The resultant amplitude of AMPA current, NMDA current and AMPA/NMDA ratio were 220.02 ± 17.08 pA, 255.41 ± 24.72 pA and 0.89 ± 0.04, respectively. As shown in Fig. 1A,C, nicotine significantly increased the amplitude of AMPAR current (P < 0.001) and AMPA/NMDA ratio (P < 0.001), but it did not cause significant change of NMDA current in PFC layer I neurons (P = 0.08, paired Student’s t-test, n = 10). To clearly identify the effect of nicotine on pure NMDA component, evoked EPSC at +40 mV was measured in the presence of AMPA antagonist CNQX (20 μM). As shown in Fig. 1B, nicotine did not cause significant changes of pure NMDA current at +40 mV, which was 136.38 ± 13.04 pA before and 132.61 ± 17.003 pA after nicotine application, respectively (P = 0.566, paired Student’s t-test; n = 8). These results suggest that nicotine significantly increases APMA current, leaving the mixed NMDA or chemically isolated NMDA current unchanged.

To further determine the detailed effect of nicotine on evoked EPSC at different holding potentials, the time course experiments were performed. As shown in Fig. 1D, after nicotine application, the evoked EPSC gradually increased and reached a steady state in 15–25 min when the holding potential was at −70, −40, −20 and +20 mV (160.20 ± 4.85%, n = 7; 141.89 ± 5.07%, n = 5; 138.01 ± 7.52%, n = 5; and 115.46 ± 3.53%, n = 5; respectively; P < 0.001 in each group, paired Student’s t-test, data were collected at 20 min after nicotine). However, nicotine treatment did not change the evoked EPSC relative to basal level at +40 or +60 mV (99.36 ± 1.37%, P = 0.26; 102.88 ± 2.31%, P = 0.30; respectively; paired Student’s t-test, n = 5 in each group). These results indicated that although nicotine proportionally increased AMPA current at negative holding potentials, the nicotine effect was smaller at +20 mV than that at −20 mV and was lost at +40 or +60 mV, suggesting that nicotine causes an inward rectification of AMPA current.

We also measured AMPA current in response to different concentrations of nicotine. As shown in Fig. 1E, the amplitudes of AMPA current were (in pA): 146.06 ± 13.41 (0 μM), 169.54 ± 16.65 (0.5 μM), 206.57 ± 20.69 (5 μM), and 206.54 ± 22.04 (50 μM), respectively. The corresponding P values were 0.03 at 0.5 μM, 0.004 at 5 μM and 0.005 at 50 μM of nicotine, respectively (paired Student’s t-test, n = 7). As shown in Fig. 1F, the AMPA/NMDA ratios were: 0.53 ± 0.04 (0 μM), 0.63 ± 0.03 (0.5 μM), 0.78 ± 0.08 (5 μM), and 0.77 ± 0.08 (50 μM), respectively. Statistical analyses showed that 0.5 μM nicotine was enough to cause a significant enhancement of AMPA/NMDA ratio, with the P values being 0.03 at 0.5 μM, 0.003 at 5 μM and 0.005 at 50 μM of nicotine, respectively (paired Student’s t-test, n = 7).

### Enhancement of AMPA current by nicotine was dependent on α7-nAChR

**Figure 2:**
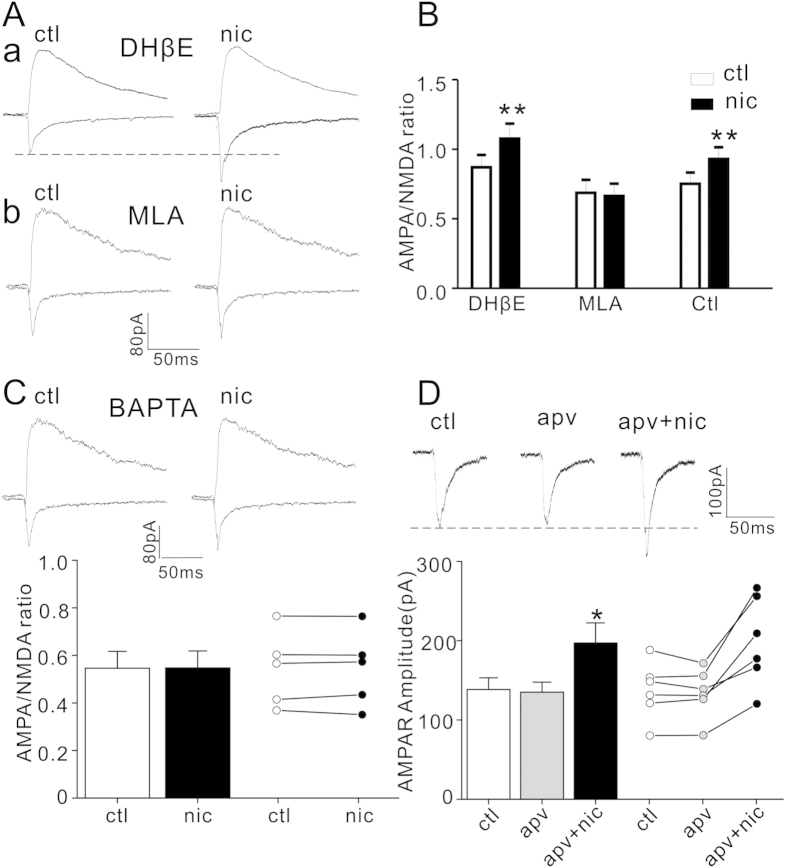
Nicotine effect on AMPA current and AMPA/NMDA ratio are dependent on α7-nAChR and intracellular calcium but not NMDA receptor. (**A**) Representative traces of AMPA and NMDA currents before (ctl) and after (nic) nicotine in slices preincubated with DHβE (1 μM, top) or MLA (10 nM, bottom); (**B**) Bar plot summary of nicotine effect on AMPA/NMDA ratio in DHβE, MLA and control (ctl). **P < 0.001 (nicotine vs. control, paired Student’s t-test, n = 6). (**C**) Representative traces of AMPA and NMDA currents before (ctl) and after (nic) nicotine treatment with intracellular infusion of BAPTA (10 mM) are shown on the top; histograms and bar plots of AMPA/NMDA ratio are shown on the bottom (P > 0.05, paired Student’s t-test, n = 5). (**D**) Representative traces (top) and histograms and dot plots (bottom) of AMPA currents before (ctl), after APV (apv, 50 μM) and APV with nicotine (apv +nic). P = 0.368, apv vs. control; *P = 0.009, apv vs. nic +apv; P = 0.013, ctl vs. nic +apv (paired Student’s t-test, n = 6).

Layer I neurons express both α7- and non α7- nicotinic receptors^13^. To determine which nAChR subtype might be responsible for nicotine-induced increase of AMPA current, slices were pre-incubated with a selective α4β2-nAChR antagonist dihydro-β-erythroidine (DHβE, 1 μM) or a selective α7-nAChR antagonist methyllycaconitine (MLA,10 nM) for one hour before recording. Figure 2A,B showed that DHβE failed to abolish nicotine-induced enhancement of AMPA/NMDA ratio (0.87 ± 0.08 before and 1.08 ± 0.10 after nicotine, p < 0.001, paired Student’s t-test, n = 7). MLA pre-incubation prevented nicotine effect on AMPA/NMDA ratios, which were 0.69 ± 0.09 before and 0.66 ± 0.09 after nicotine administration, respectively (p = 0.16, n = 6). These results suggest that nicotine increases AMPA/NMDA ratio via α7-nAChR mediated mechanism in our experimental conditions.

Activation nAChRs could promote Ca^2+^ influx^29^. Thus we tested if nicotine effect on AMPA current was Ca^2+^-dependent. As shown in Fig. 2C, pipette inclusion of calcium chelator BAPTA (10 mM) blocked nicotine enhancement of AMPA/NMDA ratio (0.55 ± 0.07 before and 0.56 ± 0.07 after nicotine, P = 0.87, paired Student’s t-test, n = 5). Previous study has indicated that systemic nicotine-induced enhancement of AMPA/NMDA ratio is dependent on NMDA receptor in dopamine neurons in the ventral tegmental area (VTA)^15^. Thus we also assessed nicotine effect in the presence of NMDAR inhibitor APV (50 μM). As shown in Fig. 2D, the amplitude of AMPA current was 138.32 ± 14.75 pA before and 135.08 ± 12.68 pA after APV treatment, respectively (P = 0.37, paired Student’s t-test, n = 6). However, subsequent application of nicotine in the presence of APV could still induce an augmentation of AMPA current (197.09 ± 25.28 pA; P = 0.009, APV +nicotine vs. APV alone, paired Student’s t-test, n = 6). These results suggest that the enhancement of AMPA current is dependent on intracellular Ca^2+^ but not NMDAR.

### Nicotine effect on mEPSC was dependent on α7-nAChR

**Figure 3:**
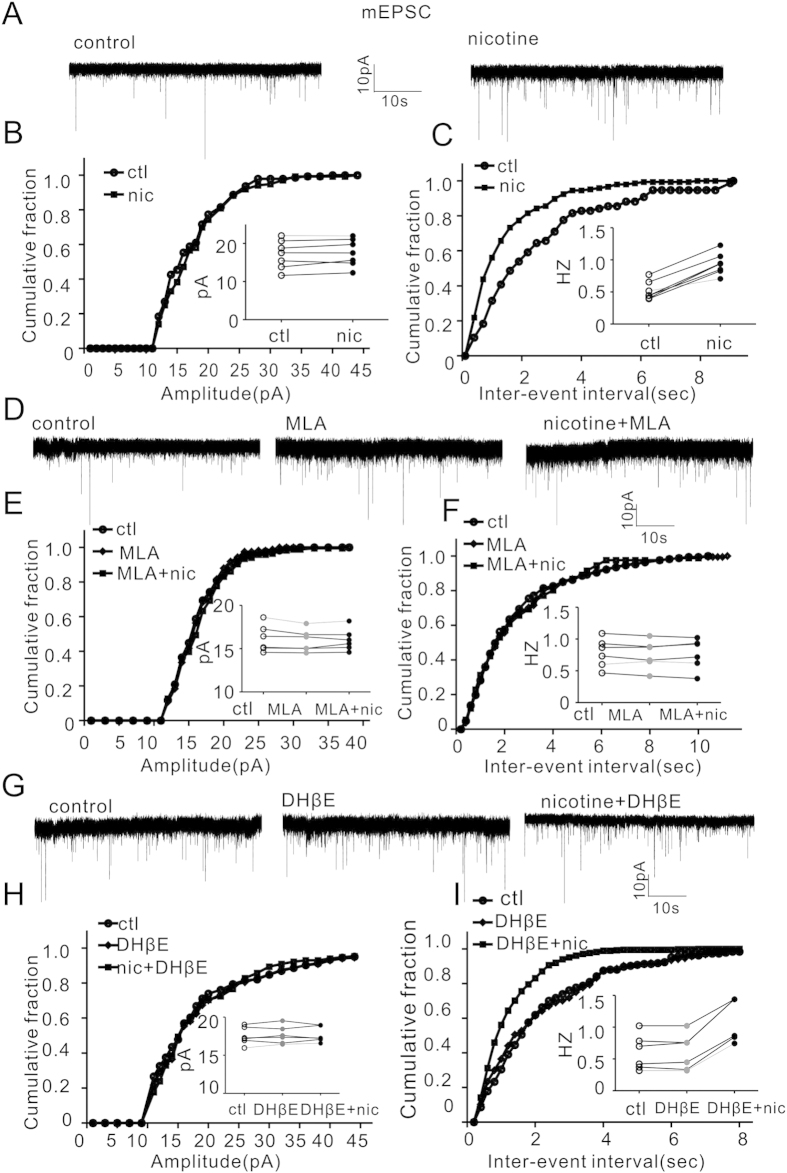
Nicotine increases mEPSC frequency via α7-nAChR. (**A**) Representative traces of mEPSC before (left) and after (right) nicotine (5 μM) application. (**B**,**C**) Cumulative fractions and dot plots (insets) of mEPSC amplitude and frequency before and after nicotine application. P < 0.001 (nicotine vs. control in the same cells, paired Student’s t-test, n = 7). (**D**) Representative traces of mEPSC before (control) and after MLA alone (10 nM) and MLA (10 nM) with nicotine (5 μM) treatment. (**E**,**F**) Cumulative fractions and dot plots (insets) of mEPSC before and after treatments shown in D (P > 0.05, two way ANOVA followed by Bonferroni’s LSD post hoc test, n = 6). (**G**) Representative traces of mEPSC before (control) and after DhβE alone (1 μM) and DhβE(1 μM) with nicotine(5 μM) treatment. (**H,I**) Cumulative fractions and dot plots (insets) of mEPSC amplitude and frequency before and after treatments in G. (P = 0.96, ctl vs. DhβE; P < 0.0001 ctl vs. DhβE +nicotine; P < 0.0001, DhβE vs. DhβE +nicotine; two way ANOVA followed by Bonferroni’s LSD post hoc test, n = 6)

To identify the role of α7-nAChR in short-term synaptic plasticity^30^, we measured nicotine effect on mEPSC in the presence of two nAChR inhibitors MLA and DhβE. As shown in Fig. 3A–C, nicotine (5 μM) bath application alone for 10 minutes increased the mEPSC frequency, which was (in Hz) 0.52 ± 0.05 before and 0.78 ± 0.04 after nicotine treatment (p < 0.001, paired Student’s t-test, n = 7). However, nicotine did not change mEPSC amplitude (17.14 ± 1.41 pA before and 17.58 ± 1.34 pA after nicotine, P = 0.19, paired Student’s t-test, n = 7). While bath application of α7-nAChR antagonist MLA (10 nM) alone for 10 min did not cause significant changes of mEPSC, co-application of MLA with nicotine diminished nicotine augmentation of mEPSC frequency (Fig. 3D–F). The amplitudes in control, MLA and MLA +nicotine were (in pA) 16.17 ± 0.63, 16.01 ± 0.44, and 16.02 ± 0.52, respectively; and the corresponding frequencies were (in Hz) 0.78 ± 0.09, 0.75 ± 0.08 and 0.77 ± 0.04, respectively (P > 0.05 in all groups, two way ANOVA followed by Bonferroni’s LSD post hoc test, n = 6). In contrast, α4β2-nAChR antagonist DhβE (1 μM) did not block nicotine effect on mEPSC frequency (Fig. 3G–I). The amplitudes in control, DhβE and DhβE +nic were (in pA) 17.52 ± 0.47, 17.66 ± 0.48 and 17.22 ± 0.42, respectively, while the frequencies (in Hz) were 0.60 ± 0.11, 0.61 ± 0.12 and 1.13 ± 0.14, respectively (P < 0.001, DhβE vs. DhβE +nic, two way ANOVA followed by Bonferroni’s LSD post hoc test, n = 5). These results suggest that α7-nAChR mediates nicotine enhancement of mEPSC frequency.

### Nicotine-mediated paired-pulse inhibition was blocked by α7-nAChR antagonist

**Figure 4:**
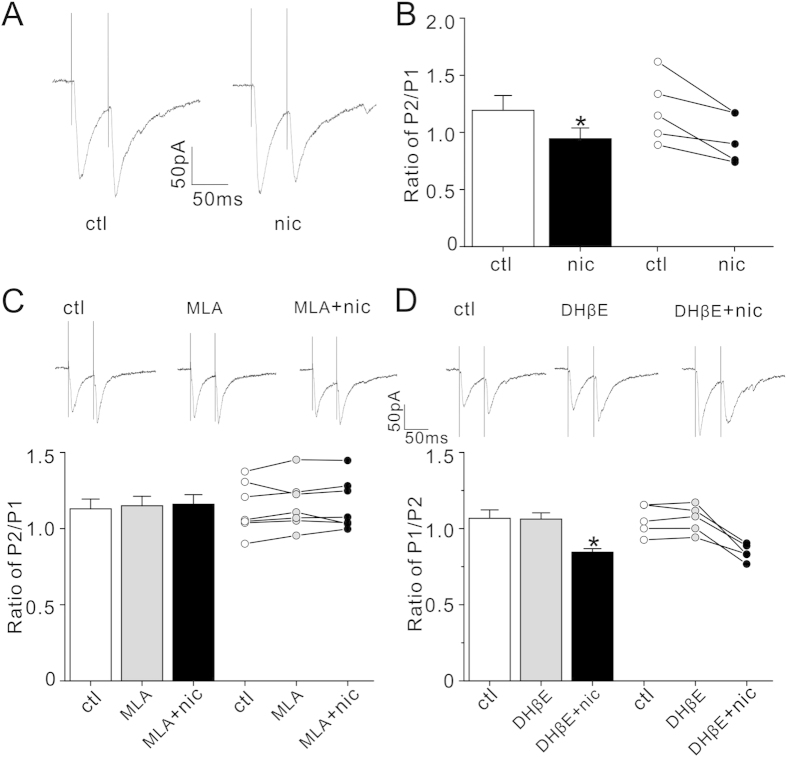
Paired-pulse inhibition by nicotine is blocked by α7-nAChR antagonist. (**A**) Representative traces resulting from paired-pulse stimulation at an interpulse interval of 50 ms in the absence (ctl) and presence nicotine (nic). (**B**) Histograms and dot plots showing nicotine treatment decreases the paired-pulse ratio (PPR, second peak over first peak, P2/P1). *p < 0.05 (nicotine vs. control in the same cells, paired Student’s t-test, n = 5). (**C**) Representative traces resulting from paired-pulse stimulation (top) with histograms and dot plots of PPR (bottom) before (ctl), after MLA alone (10nM) and MLA (10nM) with nicotine (5 μM) (P > 0.05 in all groups, two way ANOVA followed by Bonferroni’s LSD post hoc test, n = 7). (**D**) Representative traces resulting from paired-pulse stimulation (top) with histograms and dot plots of PPR (bottom) before (ctl), after DhβE alone (1 μM) and DhβE (1 μM) with nicotine (5 μM) (P = 0.90, ctl vs. DhβE; P = 0.03, ctl vs. DhβE +nic; *P = 0.04, DhβE vs. DhβE +nic, two way ANOVA followed by Bonferroni’s LSD post hoc test, n = 5).

To further determine whether presynaptic α7-nAChR mediates nicotinic augmentation of glutamate release, we performed the paired-pulse experiment. Nicotine (5 μM) perfusion only for 10 minutes decreased the paired-pulse ratio (PPR, Fig. 4A,B, p = 0.02, paired Student’s t-test, n = 5), suggesting that nicotine increased glutamate release probability. MLA (10 nM) perfusion for 10 minutes did not change the basal PPR but prevented nicotine-mediated PPR inhibition (P > 0.05 in all groups, two way ANOVA followed by Bonferroni’s LSD post hoc test, n = 7). Again, DhβE (1 μM) itself did not change the PPR (p = 0.90, DhβE vs. control, n = 5), but it failed to block the nicotine effect on PPR (P = 0.90, ctl vs. DhβE; P = 0.03, ctl vs. DhβE +nic; *P = 0.04, DhβE vs. DhβE +nic, two way ANOVA followed by Bonferroni’s LSD post hoc test, n = 5). These results suggest that α7-nAChR mediates nicotinic inhibition of PPR.

### Ca^2+^-permeable AMPARs (CP-AMPARs) were involved in nicotinic regulation of AMPA current

**Figure 5:**
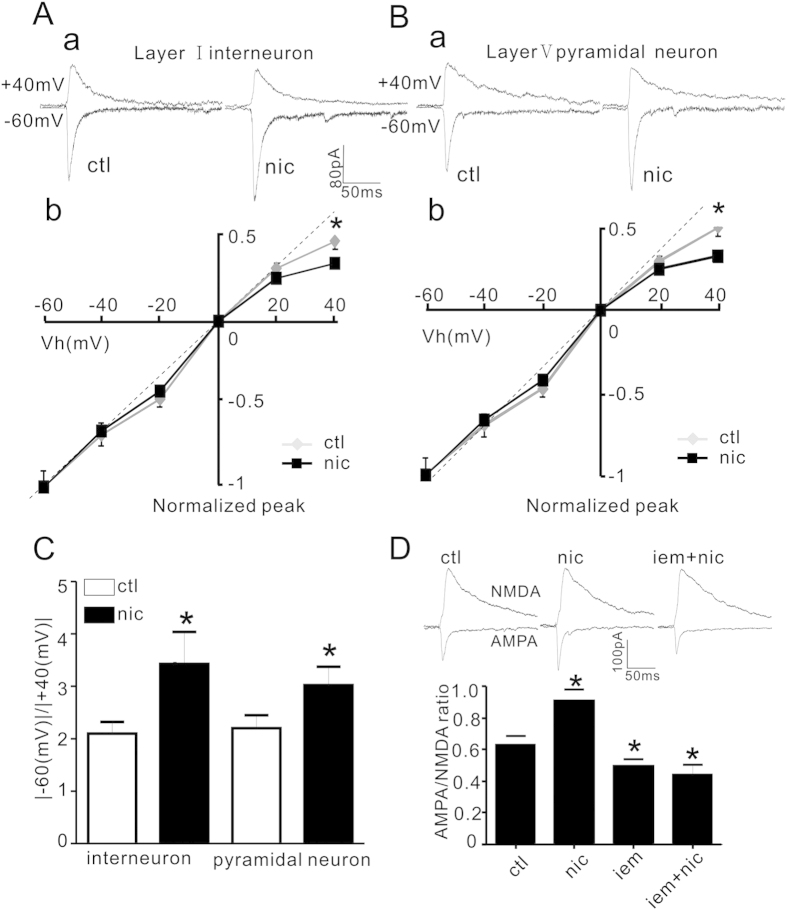
Ca2+-permeable AMPARs (CP-AMPARs) are involved in nicotine enhancement of AMPAR current. (**A**) (**a**), Representative traces of AMPA currents before (ctl) and after nicotine (nic) in layer I neurons at −60 and +40 mV (Vh); (**b**), I-V curves of AMPA currents. (**B**) (**a**), Representative traces of AMPA currents before (ctl) and after (nic) nicotine treatment in layer V pyramidal neurons at −60 and +40 mV (Vh); (**b**), I-V curves of AMPA currents in pyramidal neurons. (**C**) Bar plot summary of inward rectification (IR) index in layer I neurons and layer V pyramidal neurons, IR index is increased after nicotine treatment in lay I neurons (P = 0.032, paired Student’s t-test, control vs. nicotine in the same cells, n = 7) and layer V pyramidal neurons (P = 0.028, paired Student’s t-test, n = 6) respectively. (**D**) Representative traces (top) and bar plot summary (bottom) of AMPA and NMDA currents before (ctl) and after nicotine(5 μM), IEM-1460 (iem, 50 μM) and IEM-1460 + nicotine (iem +nic). P = 0.001, nic vs. ctl; P = 0.005 iem vs. ctl; P < 0.001 iem vs. nic; P = 0.47, IEM-1460 +nicotine vs. IEM-1460 (two way ANOVA followed by Bonferroni’s LSD post hoc test, n = 5).

AMPARs are heterotetramer composed of GluA1-A4 (also termed GluR1-R4)^24^. The GluA2-lacking AMPARs exhibit calcium permeability with characteristic inwardly rectifying current-voltage (I-V) relationship^24^,^25^,^31^,^32^. The inward rectification may reflect the insertion of intracellular GluA2-lacking (GluA1-containing)-AMPAR into postsynaptic membrane^15^,^26^. To identify if nicotine may regulate this inward rectification, we assessed nicotine effect on the I-V relationship in the presence of intracellular spermine (100μM) and extracellular NMDA antagonist APV (50 μM). As shown in Fig. 5A,C, 10 min application of nicotine caused an increase of inward rectification (IR), which was 2.09 ± 0.22 before and 3.42 ± 0.61 after nicotine (P = 0.032, paired Student’s t-test, n = 7). Interestingly, nicotine also increased the inward rectification of AMPA current in lay V pyramidal neurons (Fig. 5B,C, 2.14 ± 0.20 before and 2.89 ± 0.31 after nicotine; P = 0.028, paired Student’s t-test, n = 6). These results suggest that common mechanisms might be involved in nAChR regulation of AMPAR trafficking in layer I and layer V neurons.

To further validate that the IR was mediated by CP-AMPAR^33^,^34^, we assessed nicotine effect on AMPA/NMDA ratio in the presence of a selective CP-AMPAR inhibitor IEM-1460^35^. As shown in Fig. 5D, nicotine caused an increase of AMPA/NMDA ratio in control condition (0.63 ± 0.06 before and 0.91 ± 0.07 after nicotine, P = 0.001, two way ANOVA followed by Bonferroni’s LSD post hoc test, n = 5). Subsequent application of IEM-1460 alone (50 μM) for 10 min caused a significant reduction of AMPA/NMDA ratio (0.47 ± 0.04, P < 0.001, IEM-1460 vs. nicotine; p = 0.005, IEM-1460 vs. control, two way ANOVA followed by Bonferroni’s LSD post hoc test, n = 5). However, the effect of nicotine was absent in the presence of IEM-1460 (0.42 ± 0.05, P = 0.47, IEM-1460+ nicotine vs. IEM-1460 alone, two way ANOVA followed by Bonferroni’s LSD post hoc test, n = 5). The results suggest that nicotine enhancement of AMPAR or AMPA/ NMDA ratio is via an increase in postsynaptic CP-AMPAR.

### Inhibition of GluA1 phosphorylation sites diminished nicotine effect on AMPA current

**Figure 6:**
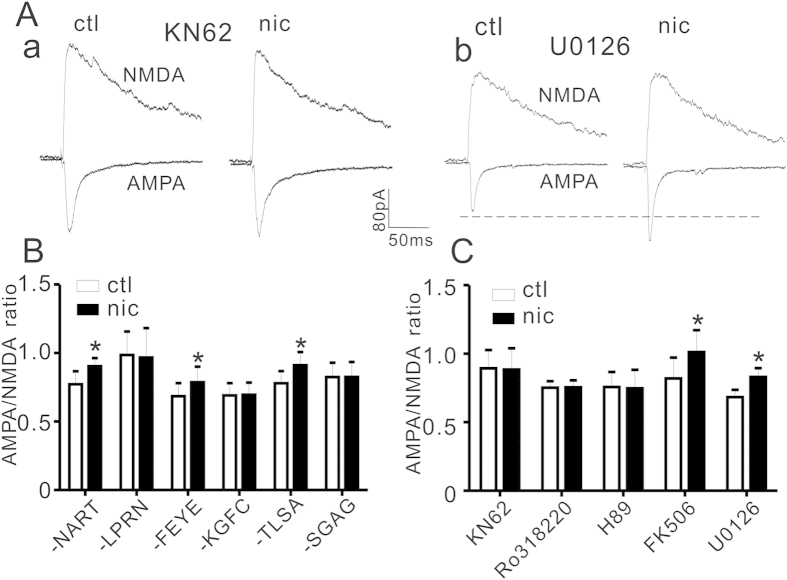
CaMKII, PKC and PKA are involved in nicotine regulation of AMPA/NMDA ratio. (**A**) Representative traces of AMPA and NMDA currents before (ctl) and after nicotine treatment (nic) in the presence of CaMKII inhibit KN62 (**a**) or ERK inhibitor U0126 (**b**). (**B**) Bar plot summary of nicotine effect on AMPA/NMDA ratio when pipette solution was infused with (all in 200 μM) CaMKII scrambled peptide –NART (n = 7), inhibitory peptide–LPRN (n = 5); PKC scrambled peptide–FFYE (n = 6), inhibitory peptide –KGFC (n = 7); PKA scrambled peptide –TLSA (n = 6) or inhibitory peptide –SGAG (n = 7), respectively. P values are <0.001, 0.19, 0.002, 0.36, <0.001 and 0.94, respectively (nicotine vs. control in the same cells, paired Student’s t-test). (**C**) Bar plot summary of nicotine effect on AMPA/NMDA ratio when pipette solution was infused with CaMKII inhibitor KN-62 (15 μM, n = 6), PKC inhibitor Ro318220 (10 μM, n = 7), PKA inhibitor H89 (10 μM, n = 7), CN inhibitor FK506 (10 μM, n = 6) or ERK inhibitor U0126 (10 μM, n = 8). P values are 0.85, 0.29, 0.59, 0.003 and <0.001, respectively (nicotine vs. control in the same cells, paired Student’s t-test).

Previous study demonstrates that intracellular C-terminal of GluA1 contains CaMKII, PKC and PKA phosphorylation sites, which might regulate GluA1 membrane insertion^32^. In addition, synthetic peptides designed to block GluA1 at these phosphorylation sites have been shown to regulate GluA1 cell surface expression^36^. Thus we tested if these peptides regulate nicotine effect on AMPA current. As shown in Fig. 6A,B, pipette inclusion of CaMKII peptide -LPRN significantly reduced nicotine effect on AMPA/NMDA ratio (0.98 ± 0.17 before and 0.97 ± 0.21 after nicotine, P = 0.19, paired Student’s t-test, n = 5) when compared with control peptide-NART (0.77 ± 0.09 before and 0.90 ± 0.06 after nicotine, P < 0.001, paired Student’s t-test, n = 7). Similarly, nicotine enhancement of AMPA/NMDA ratio was significantly diminished after intracellular application of PKC or PKA peptide. The AMPA/NMDA ratios were 0.69 ± 0.09 before and 0.70 ± 0.09 after nicotine in PKC peptide -KGFC (P = 0.36, paired Student’s t-test, n = 7); 0.68 ± 0.10 before and 0.79 ± 0.11 after nicotine in PKC control peptide -FFYE(P = 0.002, paired Student’s t-test, n = 6); 0.84 ± 0.10 before and 0.85 ± 0.11 after nicotine in PKA peptide –SGAG (P = 0.94, paired Student’s t-test, n = 7) and 0.78 ± 0.09 before and 0.91 ± 0.10 after nicotine (P < 0.001, paired Student’s t-test, n = 6) in PKA control peptide -TLSA. These results suggest that nicotinic regulation of AMPAR current is dependent on GluA1 phosphorylation state.

To further test if nicotine effect is regulated by protein kinases acting on GluA1, intracellular inclusion of corresponding protein kinase inhibitor was applied. As shown in Fig. 6C, CaMKII inhibitor KN-62(15 μM) significantly blocked the enhancement of AMPA/NMDA by nicotine (0.89 ± 0.13 before and 0.89 ± 0.15 after nicotine, p = 0.85, paired Student’s t- test, n = 6). Also, nicotine regulation of APMA/NMDA ratio was significantly inhibited in the presence of PKC inhibitor Ro-318220 (10 μM) or PKA inhibitor H-89 (10 μM). The AMPA/NMDA ratios were 0.75 ± 0.11 before and 0.74 ± 0.13 after nicotine in Ro-318220 (P = 0.29, paired Student’s t- test, n = 7) and 0.74 ± 0.05 before and 0.75 ± 0.05 after nicotine (P = 0.59, paired Student’s t-test, n = 7).

ERK and calcineurin (CN) are two important molecules in nAChR signaling^37^. Thus we also tested the effect of their inhibitors on nicotine regulation of AMPA current. However, neither ERK inhibitor U0126 (10 μM) nor CN inhibitor FK506 (10 μM) was able to block nicotine enhancement of AMPA/NMDA ratios (Fig. 6C), which were 0.68 ± 0.05 before and 0.83 ± 0.06 after nicotine in U0126 (P < 0.001, paired Student’s t-test n = 8); and 0.82 ± 0.15 before and 1.01 ± 0.16 after nicotine in FK506 (P = 0.003, paired Student’s t-test, n = 6). These results indicated that ERK and CN were not involved in nicotinic regulation of AMPA current.

## Discussion

The current study has shown that nicotine selectively increases the AMPA current in PFC layer I neurons. This effect is blocked by α7-nAChR antagonist and is dependent on intracellular calcium. The characteristic inward rectification of CP-AMPAR suggests an involvement of GluA1 in nicotine augmentation of AMPAR current, which is validated by the use of synthetic peptides that are designed to block phosphorylation sites of GluA1.

The present study provides evidence that nAChR causes an increase in AMPAR current but not NMDAR current in layer I neurons. The unresponsiveness of NMDAR current in layer I is in contrast to what has been found in pyramidal neurons, in which nAChR activation causes a sustained reduction of NMDAR current involving ER calcium store and ERK activation^16^. Since almost all neurons in layer I are GABAergic^8^, our results suggest that different mechanisms might underlie NMDAR regulation between inhibitory and excitatory neurons. Indeed, ERK or calcineurin inhibitor does not block nicotinic regulation of AMPA current (Fig. 6C). With respect to AMPAR, a consistent response seems to be existed in both layer I and layer V pyramidal neurons (Fig. 5). Consistently, a recent study has shown that nicotine facilitates the expression of GluA1 containing AMPAR in hippocampal neurons^38^.

In layer I neurons, the mRNAs of α4β2- and α7-nAChR are predominantly expressed^13^. And the selective antagonist of α4β2- or α7-nAChR blocks nicotine enhancement of neuronal excitation in layer I neurons^13^,^14^, suggesting that both receptor subtypes mediate nicotinic regulation of neuronal activity in layer I. In our study however, the enhancement of AMPAR by nicotine is blocked by MLA but not DHβE, suggesting that α7-nAChR is crucial (Fig. 2B). It is reasonable that activation of α7-nAChR might promote Ca^2+^ influx and trigger Ca^2+^-dependent cellular processes including synaptic plasticity, neurotransmitter release, cell migration, and survival^39^,^40^. Consistently, intracellular inclusion of calcium chelator blocks nicotinic effect on AMPA current. However, this effect cannot be inhibited by NMDAR inhibitor (Fig. 2). Previous study has shown that NMDAR antagonist prevents systemic nicotine induced increase of AMPA/NMDA ratio in dopaminergic ventral tegmental area (VTA)^15^. In their study, increased AMPA/NMDA ratio in VTA neurons occurs in 1 hour later and lasts for 3 days, but not in 10 minutes after a single systemic administration of nicotine. And the NMDAR antagonist MK-801 is also injected intraperitoneally, which is different from our experimental condition. The exact mechanisms underlying these differences are currently unknown.

In our study, nicotine effect on mEPSC frequency and PPR is blocked by MLA but not DhβE, supporting a presynaptic role of α7-nAChR. However, in the case of AMPAR regulation, the following might also suggest a postsynaptic function of α7-nAChR. First, ultrastructural study reveals that at least in the hippocampus, α7-nAChR is also located at dendritic spines in both GABAergic and glutamatergic neurons^30^. Second, the intracellular inclusion of calcium chelator, as well as GluA1 peptides, prevents nicotinic regulation of AMPA current, indicating postsynaptic events. This is further supported by the characteristic inward rectification of AMPA current, which reflects postsynaptic APMAR trafficking^15^,^26^,^31^. Finally, a recent study provides a direct evidence that postsynaptic α7-nAChR contributes to GluA1 containing AMPAR accumulation on dendritic spines in the cultured hippocampal neurons^38^. Although it is a general consensus that mEPSC frequency reflects presynaptic release probability^41^,^42^, postsynaptic AMPAR might also play a role. Béïque and colleagues have shown that in PSD-95 (post-synaptic density 95) transfected organotypic brain slices, a larger AMPA/NMDA ratio is accompanied by increased AMPAR- mediated EPSC frequency but not amplitude, whereas the PPR is without change, suggesting no alterations of presynaptic release probability^43^. This could be explained by increased number of synapse rather than increased number or function of APMAR. The same research group demonstrates that PSD-95 KO mice exhibit greater occurrence of silent synapse^44^. It might be possible in our experiment that some of the neurons in layer I are silent under normal condition, which are then activated by nicotine after synaptic insertion of GluA1 containing AMPARs, resulting in increased frequency but not amplitude of mEPSC.

It is well documented that GluA1 contains phosphorylation sites of CAMKII, PKC and PKA, which regulate its synaptic insertion, while GluA2 trafficking is linked to NSF and adaptor protein AP2, among others^31^,^32^. Consistently, these kinase inhibitors as well as inhibitory peptides that block nicotine effect on AMPA/NMDA ratios further support a role of GluA1(Fig. 6). Therefore, our study suggests that nicotine controls AMPAR current by regulating GluA1 phosphorylation state. Although PKC site is also seen GluA2, this phosphorylation leads to GluA2 internalization rather than synaptic insertion^45^.

Nicotinic effect on mEPSC (the action potential is inhibited) excludes the postsynaptic involvement of L-type voltage-dependent calcium channels; and nicotinic effect on AMPA current is not prevented by NMDAR inhibitor, which excludes other major calcium entry mediators that might play important role in this regulation. Therefore, we propose that nAChR activation through α7-nAChR results in increased intracellular calcium, which in turn might activate CAMKII and PKC; and the resultant increase in phosphorylation of GluA1 leads to enhanced synaptic insertion and AMPAR current in layer I neurons. PKA phosphorylation of GluA1 seems to help “prime” GluA1 containing AMPARs for LTP, thus might maintain their retention at synaptic sites^46^. However, despite that PKA contributes to α7-nAChR enhancement of glutamatergic neurotransmission^47^, direct evidence that links nAChR and PKA activation is sparse. On the other hand, inhibitors of calcineurin and ERK, two important nAChR downstream signaling molecules, fail to block nicotine regulation of AMPAR current (Fig. 6), suggesting that calcineurin and ERK do not play a role in this case. Rather, ERK^16^ and calcineurin^48^ might be involved in NMDAR regulation.

### Clinical significance

AMPAR trafficking is involved in synaptic remodeling, such as long-term potentiation and depression. Growing evidence suggests that AMPAR-dependent synaptic plasticity is closely related to hippocampus- and amygdale-dependent learning and memory^49^. It is known that addictive drugs can cause behavioral alterations through AMPAR trafficking in the mesolimbic reward system including PFC and the ventral tegmetal area^5^,^49^. And the role of nicotine in cognition by acting on PFC networks has been well-documented^3^,^4^. Therefore, nicotinic regulation of AMPAR trafficking in layer I in our study might provide an important mechanism underlying nicotine related behaviors.

## Additional Information

**How to cite this article**: Tang, B. *et al*. Modulation of AMPA receptor mediated current by nicotinic acetylcholine receptor in layer I neurons of rat prefrontal cortex. *Sci. Rep*. **5**, 14099; doi: 10.1038/srep14099 (2015).
