# Corrigendum: Modulation of AMPA receptor mediated current by nicotinic acetylcholine receptor in layer I neurons of rat prefrontal cortex

**DOI:** 10.1038/srep41465

**Published:** 2017-02-06

**Authors:** Bo Tang, Dong Luo, Jie Yang, Xiao-Yan Xu, Bing-Lin Zhu, Xue-Feng Wang, Zhen Yan, Guo-Jun Chen

Scientific Reports 5: Article number: 1409910.1038/srep14099; published online: 09 15 2015; updated: 02 06 2017

The authors of Article made an error in preparation of the final version of Figure 2 and inadvertently used panel 2C as both panel 2Ab and panel 2C. A revised version of Figure 2 with the correct panel 2Ab is published below. Other panels of this figure are unchanged.

This correction does not affect the conclusions of the Article. The authors apologize for the error and any inconvenience caused by it.

## Figures and Tables

**Figure 2:**
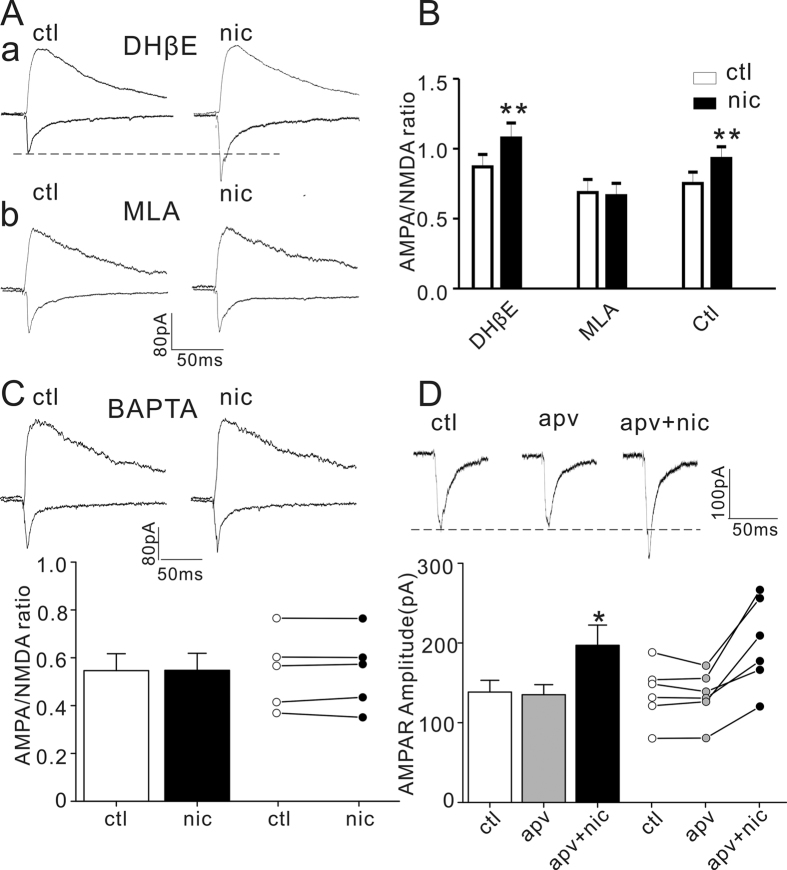
Nicotine effect on AMPA current and AMPA/NMDA ratio are dependent on α7-nAChR and intracellular calcium but not NMDA receptor. (**A**) Representative traces of AMPA and NMDA currents before (ctl) and after (nic) nicotine in slices preincubated with DHβE (1 μM, top) or MLA (10 nM, bottom); (**B**) Bar plot summary of nicotine effect on AMPA/NMDA ratio in DHβE, MLA and control (ctl). **P < 0.001 (nicotine vs. control, paired Student’s t-test, n = 6). (**C**) Representative traces of AMPA and NMDA currents before (ctl) and after (nic) nicotine treatment with intracellular infusion of BAPTA (10 mM) are shown on the top; histograms and bar plots of AMPA/NMDA ratio are shown on the bottom (P > 0.05, paired Student’s t-test, n = 5). (**D**) Representative traces (top) and histograms and dot plots (bottom) of AMPA currents before (ctl), after APV (apv, 50 μM) and APV with nicotine (apv +nic). P = 0.368, apv vs. control; *P = 0.009, apv vs. nic +apv; P = 0.013, ctl vs. nic +apv (paired Student’s t-test, n = 6).

